# HPV genotyping and risk factors for anal high-risk HPV infection in men who have sex with men from Toronto, Canada

**DOI:** 10.1038/s41598-021-84079-y

**Published:** 2021-02-26

**Authors:** Yoojin Choi, Mona Loutfy, Robert S. Remis, Juan Liu, Anuradha Rebbapragada, Sanja Huibner, Jason Brunetta, Graham Smith, Tatjana Reko, Roberta Halpenny, Rupert Kaul, Troy Grennan

**Affiliations:** 1 Department of Immunology, University of Toronto, Toronto, Ontario Canada; 2 Dalla Lana School of Public Health, University of Toronto, Toronto, Ontario Canada; 3 Maple Leaf Medical Clinic, Toronto, Ontario Canada; 4 Women’s College Research Institute, Women’s College Hospital, Toronto, Ontario Canada; 5 Public Health Laboratory-Toronto, Public Health Ontario, Toronto, Ontario Canada; 6 Canada HealthLabs, Toronto, Ontario Canada; 7 Department of Medicine, University of Toronto, Toronto, Ontario Canada; 8 GlaxoSmithKline, Mississauga, Ontario Canada; 9 Department of Medicine, University Health Network, Toronto, Ontario Canada; 10 BC Centre for Disease Control, Vancouver, British Columbia Canada; 11 Department of Medicine, University of British Columbia, Vancouver, British Columbia Canada; 12 St. Paul’s Hospital, Vancouver, British Columbia Canada

**Keywords:** Diseases, Risk factors

## Abstract

Men who have sex with men (MSM) are disproportionately affected by anal cancer, predominantly caused by high-risk (HR) human papillomavirus (HPV) infection. Currently, the nonavalent HPV vaccine provides coverage against nine HPV genotypes, including seven HR-HPV genotypes. Here, we characterize anal HR-HPV genotype distribution and associated risk factors in MSM from Toronto, Canada recruited between September 2010 and June 2012. Wilcoxon–Mann–Whitney test was used for continuous variables, Chi-square test was performed for categorical variables, and a multivariable model using logistic regression was created to assess for correlates of anal HR-HPV infection. A total of 442 MSM were recruited, with a median age of 45 (IQR 38–50) and an overall HPV prevalence of 82%. The prevalence of any HR-HPV infection was 65.3% and 50.7% in the HIV-positive and HIV-negative MSM, respectively. No participant tested positive for all genotypes covered by the nonavalent vaccine. HIV status (aOR 1.806; 95% CI 1.159–2.816), smoking (aOR 2.176; 95% CI 1.285–3.685) and the number of lifetime sexual partners (aOR 2.466; 95% CI 1.092–5.567) were independent risk factors for anal HR-HPV infection. Our findings will be useful to inform HPV vaccine rollout and HPV prevention strategies in Canadian MSM.

## Introduction

Human papillomavirus (HPV) is the most common sexually transmitted infection (STI) globally^1^. Of the more than 200 HPV genotypes that have been identified to date, over 50 genotypes primarily target the transformation zone between the squamous and columnar epithelia of mucosal surfaces such as the ano-rectum^2,3^. Importantly, while most mucosal HPV infections have no symptoms and are spontaneously cleared by the host immune system, some cause persistent infection and can eventually lead to cancer of the affected area^4^. Mucosal HPV genotypes are classified as either low-risk (LR) or high-risk (HR), with LR genotypes sometimes causing benign warts but not cancerous lesions, while HR genotypes have oncogenic potential^5^.

Anal cancer is an important consequence of HPV infection, with a meta-analysis finding over 80% of anal cancers to be linked to HPV infection, most strongly with HPV-16 at a rate of 82% in HIV-negative and 67% in HIV-positive men^6,7^. The incidence of anal cancer has been increasing in high-income countries^8^, with particularly rapid increases in men who have sex with men (MSM) where the incidence is 40/100,000 in HIV-negative MSM and over 100/100,000 in HIV-positive MSM^9^, in comparison to a background rate of 2/100,000 in the general population^10^. Current treatment options for anal dysplasia include mechanical or chemical ablative techniques, but there are high recurrence rates regardless of clinical approach and it has not been formally demonstrated that any of these approaches prevents the subsequent development of cancer^11,12^. Moreover, recent evidence shows that the clearance of anal dysplasia in the absence of any intervention occurs at a fairly high rate of 22/100 person-years, further adding to the complexity of this clinical issue^13^.

Although clinical trials are underway to optimize screening and treatment protocols for anal HPV-associated disease, prophylactic vaccination is an important and validated method of HPV prevention. There are currently three commercially available vaccines in Canada, two of which are recommended for MSM. The quadrivalent Gardasil vaccine, which targets HPV 6, 11, 16 and 18, was shown to be effective in reducing the incidence of anal precancer^14^. More recently the nonavalent Gardasil-9 vaccine, which adds protection against HPV 31, 33, 45, 52 and 58, has also shown safety and efficacy in MSM^15^. Notably, while the HPV vaccine is recommended up to the age of 45 for everyone in the United States, the indications in Canada are only up to the age of 26 for men^16^. Nonetheless, Canada’s National Advisory Committee on Immunization (NACI) still recommends that HPV vaccine be strongly considered in all MSM and people living with HIV regardless of their age^17,18^. As such, these guidelines lead to uncertainty and inconsistency in practices around HPV vaccine recommendations^19^. Furthermore, men generally have lower acquired immunity in response to natural HPV infection as compared to women^20,21^, and unlike cervical HPV infection which peaks shortly after sexual debut, anal HPV infection in men are found at relatively constant rates across the lifespan^22,23^. Taken together, these findings suggest that older MSM may also benefit from the HPV vaccine.

Given this rationale for HPV vaccination among all MSM including older MSM, understanding the prevalence and risk factors for HR-HPV infection in this population may better inform vaccination policy, research priorities and future clinical implementation strategies. The current study expands on a prior analysis of HPV infection in MSM from Toronto^24^ to characterize genotype distribution of anal HPV infection, and risk factors associated with HR-HPV infection among HIV-positive and HIV-negative older MSM.

## Results

### Participant characteristics

A total of 442 MSM were enrolled in our study, consisting of 294 HIV-positive MSM and 148 HIV-negative MSM. The median age for the overall population was 45 (IQR 38–50), with the majority being white (74.1%). A third (31.0%) of the population had never smoked cigarettes, while 34.3% reported as former smokers and 34.7% as current smokers. Complete data including socio-demographic characteristics were available for 429 participants, of whom 267 (62.2%) tested positive for at least one high-risk (HR) HPV genotype versus 162 (37.8%) who tested negative for any HR-HPV genotype (Table 1). Demographic characteristics were broadly similar in the HR-HPV-positive and HR-HPV-negative groups.

**Table 1:** Study population characteristics by HR-HPV status. *IQR* interquartile range. ^a^Composite means any of: gonorrhea, chlamydia or syphilis. Total datapoints for each corresponding variable are indicated. Missing data from the questionnaire were due to either “don’t know” or “refuse to answer” as the response. Some laboratory data were missing for some participants.

	HR-HPV(+) HIV(+) (n = 192)	HR-HPV(+) HIV(−) (n = 75)	HR-HPV(−) HIV(+) (n = 92)	HR-HPV(−) HIV(−) (n = 70)
**Age median (IQR) (n = 191 vs 74 vs 92 vs 69)**				
	45 (37–49)	44 (35–51)	47 (40–53)	44 (37–51)
**Region of birth n (%) (n = 192 vs 75 vs 92 vs 70)**				
Canada	144 (75.0)	62 (82.7)	68 (73.9)	48 (68.6)
United States	4 (2.1)	0 (0.0)	1 (1.1)	0 (0.0)
Central or South America, Caribbean	26 (13.5)	6 (8.0)	11 (12.0)	8 (11.4)
Europe	11 (5.7)	5 (6.7)	7 (7.6)	6 (8.6)
Other	7 (3.6)	2 (2.7)	5 (5.4)	8 (11.4)
**Ethnicity (n = 185 vs 72 vs 86 vs 69)**				
White	142 (76.8)	62 (86.1)	60 (69.8)	54 (78.3)
Black	14 (7.6)	1 (1.4)	6 (7.0)	2 (2.9)
Asian	3 (1.6)	3 (4.2)	6 (7.0)	7 (10.1)
Latin American	12 (6.5)	2 (2.8)	2 (2.3)	5 (7.2)
Aboriginal	9 (4.9)	1 (1.4)	8 (9.3)	0 (0.0)
Mixed	5 (2.7)	3 (4.2)	4 (4.7)	1 (1.4)
**Education (n = 192 vs 75 vs 92 vs 70)**				
No education	0 (0.0)	0 (0.0)	0 (0.0)	0 (0.0)
Some primary school	1 (0.5)	0 (0.0)	0 (0.0)	0 (0.0)
Completed primary school	2 (1.0)	0 (0.0)	1 (1.1)	0 (0.0)
Some high school	21 (10.9)	1 (1.3)	3 (3.3)	0 (0.0)
Completed high school	21 (10.9)	5 (6.7)	13 (14.1)	5 (7.1)
Some college/university	50 (26.0)	18 (24.0)	28 (30.4)	16 (22.9)
Completed college/university	80 (41.7)	37 (49.3)	33 (35.9)	32 (45.7)
Some graduate education	9 (4.7)	5 (6.7)	7 (7.6)	6 (8.6)
Completed graduate education	8 (4.2)	9 (12.0)	7 (7.6)	11 (15.7)
**Marital status (n = 192 vs 74 vs 92 vs 70)**				
Married (female partner)	1 (0.5)	1 (1.4)	1 (1.1)	0 (0.0)
Married (male partner)	13 (6.8)	6 (8.1)	5 (5.4)	10 (14.3)
Common-law (female partner)	0 (0.0)	0 (0.0)	0 (0.0)	0 (0.0)
Common-law (male partner)	20 (10.4)	21 (28.4)	17 (18.5)	17 (24.3)
Divorced (female partner)	9 (4.7)	0 (0.0)	2 (2.2)	1 (1.4)
Divorced (male partner)	6 (3.1)	1 (1.4)	0 (0.0)	0 (0.0)
Separated (female partner)	1 (0.5)	1 (1.4)	1 (1.1)	3 (4.3)
Separated (male partner)	19 (9.9)	4 (5.4)	9 (9.8)	7 (10.0)
Widowed (female partner)	1 (0.5)	1 (1.4)	0 (0.0)	0 (0.0)
Widowed (male partner)	2 (1.0)	3 (4.1)	4 (4.3)	2 (2.9)
Single and never married	120 (62.5)	36 (48.6)	53 (57.6)	30 (42.9)
**Lifetime number of male sexual partners (n = 188 vs 74 vs 91 vs 70)**				
1–19	8 (4.3)	4 (5.4)	7 (7.7)	13 (18.6)
20–49	21 (11.2)	9 (12.2)	15 (16.5)	13 (18.6)
50 or more	159 (84.6)	61 (82.4)	69 (75.8)	44 (62.9)
**Number of male sexual partners in preceding 6 months (n = 189 vs 75 vs 92 vs 70)**				
None	31 (16.4)	6 (8.0)	29 (31.5)	6 (8.6)
1–19	130 (68.8)	51 (68.0)	53 (57.6)	50 (71.4)
20 or more	28 (14.8)	18 (24.0)	10 (10.9)	14 (20.0)
**History of receptive anal intercourse in preceding 6 months (n = 192 vs 75 vs 92 vs 70)**				
No	89 (46.4)	31 (41.3)	55 (59.8)	34 (48.6)
Yes	103 (53.6)	44 (58.7)	37 (40.2)	36 (51.4)
**Given money for sex (n = 190 vs 75 vs 90 vs 70)**				
No	174 (91.6)	69 (92.0)	84 (93.3)	63 (90.0)
Yes	16 (8.4)	6 (8.0)	6 (6.7)	7 (10.0)
**Received money for sex (n = 190 vs 75 vs 90 vs 70)**				
No	173 (91.1)	71 (94.7)	86 (95.6)	67 (95.7)
Yes	17 (8.9)	4 (5.3)	4 (4.4)	3 (4.3)
**Circumcision status (n = 191 vs 75 vs 91 vs 70)**				
No	73 (38.2)	26 (34.7)	30 (33.0)	25 (35.7)
Yes	118 (61.8)	49 (65.3)	61 (67.0)	45 (64.3)
**Alcohol intake (n = 192 vs 74 vs 92 vs 70)**				
Never	25 (13.0)	7 (9.5)	14 (15.2)	7 (10.0)
Less than once a month	48 (25.0)	9 (12.2)	19 (20.7)	6 (8.6)
Once a month	19 (9.9)	4 (5.4)	7 (7.6)	1 (1.4)
2–3 times a month	21 (10.9)	14 (18.9)	14 (15.2)	13 (18.6)
Once a week	13 (6.8)	8 (10.8)	10 (10.9)	14 (20.0)
2–3 times a week	45 (23.4)	18 (24.3)	15 (16.3)	16 (22.9)
4–6 times a week	10 (5.2)	11 (14.9)	3 (3.3)	8 (11.4)
Daily	11 (5.7)	3 (4.1)	7 (10.9)	5 (7.1)
**Smoking history (n = 192 vs 75 vs 92 vs 70)**				
Never	52 (27.1)	19 (25.3)	31 (33.7)	31 (44.3)
Former	56 (29.2)	32 (42.7)	36 (39.1)	23 (32.9)
Current	84 (43.8)	24 (32.0)	25 (27.2)	16 (22.9)
**Recreational drug use in preceding 6 months (n = 189 vs 74 vs 92 vs 69)**				
No	63 (33.3)	23 (31.1)	35 (38.0)	33 (47.8)
Yes	126 (66.7)	51 (68.9)	57 (62.0)	36 (52.2)
**Prior history of bacterial STIs (self-reported)**				
Gonorrhea (n = 190 vs 74 vs 91 vs 65)	107 (56.3)	22 (29.7)	45 (49.5)	21 (32.3)
Chlamydia (n = 185 vs 73 vs 87 vs 66)	62 (33.5)	15 (20.5)	21 (24.1)	13 (19.7)
Syphilis (n = 189 vs 72 vs 90 vs 67)	75 (40.0)	17 (23.6)	26 (28.9)	10 (14.9)
Composite^a^ (n = 191 vs 75 vs 92 vs 69)	135 (70.7)	33 (44.0)	63 (68.5)	31 (44.9)
**Current bacterial STIs (diagnosed at study visit)**				
Gonorrhea (n = 191 vs 75 vs 91 vs 70)	0 (0.0)	1 (1.3)	0 (0.0)	0 (0.0)
Chlamydia (n = 191 vs 75 vs 91 vs 70)	1 (0.5)	0 (0.0)	1 (1.1)	0 (0.0)
Syphilis (n = 189 vs 75 vs 92 vs 70)	24 (12.7)	3 (4.0)	6 (6.5)	2 (2.9)
Composite^a^ (n = 192 vs 75 vs 92 vs 70)	25 (13.0)	3 (4.0)	7 (7.6)	2 (2.9)

### Anal HPV prevalence by HIV status

**Figure 1:**
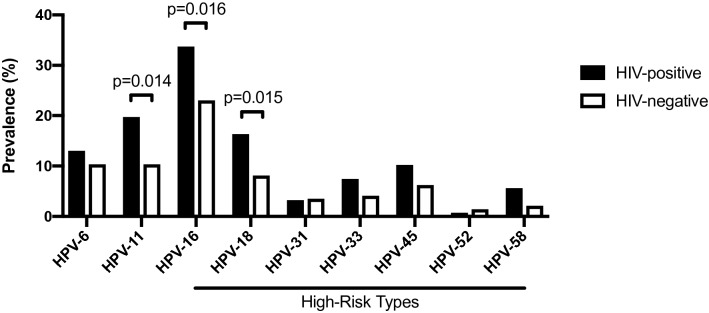
Genotype-specific prevalence of anal HPV infection in the HIV-positive (n = 294) and HIV-negative participants (n = 148).

Overall, any HPV infection and any HR-HPV infection were both commonly found in our study cohort of 442 MSM regardless of HIV status, with overall prevalence of 82.4% and 60.4%, respectively. However, MSM living with HIV were disproportionately impacted by HR-HPV infection in a number of different ways: HIV infection was associated with an increased prevalence of any HPV infection (88.4%, 95% CI [84.1–91.6%] vs 77.9% [70.5–83.9%]; p = 0.0043), any HR-HPV infection (65.3% [59.7–70.5%] vs 50.7% [42.7–58.6%]; p = 0.0013) and infection by multiple HR-HPV genotypes (29.2% [24.3–34.8%] vs 13.8% [9.1–20.3%]; p = 0.0004). Notably, HIV-positive MSM had an increased prevalence of both HPV-16 (33.7% [28.5–39.3%] vs 23.0% [16.9–30.4%]; p = 0.016) and HPV-18 (16.3% [12.5–21.0%] vs 8.1% [4.7–13.6%]; p = 0.015), as well as the low-risk HPV genotype HPV-11 that is typically associated with anogenital warts (19.7% [15.5–24.7%] vs 10.3% [6.3–16.3%]; p = 0.014; Fig. 1). Several other HR-HPV genotypes were relatively common in our cohort, regardless of HIV status. Importantly, only three participants (two living with HIV, and one not) were infected with all four HPV genotypes that are targeted by the quadrivalent vaccine (HPV-6, 11, 16 and 18) while no participant was infected by all nine genotypes targeted by the nonavalent vaccine (HPV-6, 11, 16, 18, 31, 33, 45, 52 and 58).

### Factors associated with anal HR-HPV infection

Using a priori inclusion of HIV serostatus, lifetime number of sexual partners and smoking and change-in-estimate methods to construct the multivariable model, the following variables were retained in the final multivariable analysis (Table 2): HIV-positive status; a history of receptive anal intercourse (RAI) in the previous six months; current smoking; ≥ 50 lifetime sexual partners; ≥ 5 sexual partners in the previous six months; a history of crystal methamphetamine, ecstasy or MDMA use in the previous six months, and a history of drug use within two hours of sexual activity. In the multivariable analysis, HIV infection (adjusted odds ratio [aOR] 1.806, p = 0.009), current smoking (aOR 2.176, p = 0.004) and ≥ 50 lifetime sexual partners (aOR 2.466, p = 0.030) remained significantly associated with anal HR-HPV infection (Table 2).

**Table 2:** Multivariable analysis of risk factors for anal HR-HPV infection. *aOR *adjusted odds ratio, *CI *confidence interval, Hosmer–Lemeshow goodness-of-fit statistics, p = 0.777.

	aOR	95% CI	p value
**All participants**			
Receptive anal intercourse in the last 6 months	1.472	0.927–2.338	0.101
Current smoking	2.176	1.285–3.685	**0.004**
≥ 50 lifetime number of sexual partners	2.466	1.092–5.567	**0.030**
≥ 5 number of sexual partners in the last 6 months	1.178	0.718–1.932	0.517
Use of methamphetamine/ecstasy/MDMA in the last 6 months	1.501	0.837–2.692	0.173
Drug use within 2 h of sex	0.914	0.555–1.506	0.724
HIV status	1.806	1.159–2.816	**0.009**
**HIV-positive participants**			
Receptive anal intercourse in the last 6 months	1.615	0.889–2.934	0.116
Current smoking	2.113	1.088–4.103	**0.027**
≥ 50 lifetime number of sexual partners	1.778	0.584–5.410	0.311
≥ 5 number of sexual partners in the last 6 months	1.279	0.677–2.416	0.448
Use of methamphetamine/ecstasy/MDMA in the last 6 months	1.464	0.702–3.054	0.310
Drug use within 2 h of sex	0.826	0.437–1.562	0.557
**HIV-negative participants**			
Receptive anal intercourse in the last 6 months	1.148	0.528–2.495	0.727
Current smoking	2.240	0.900–5.574	0.083
≥ 50 lifetime number of sexual partners	3.809	1.051–13.807	**0.042**
≥ 5 number of sexual partners in the last 6 months	0.983	0.433–2.233	0.968
Use of methamphetamine/ecstasy/MDMA in the last 6 months	1.777	0.657–4.810	0.258
Drug use within 2 h of sex	0.995	0.436–2.271	0.991

P value of < 0.05 was determined as statistically significant (bolded).

We then constructed multivariable models stratified by HIV status. Among HIV-positive MSM (Table 2), current smoking (aOR 2.113, p = 0.027) was the only factor that remained significantly associated with anal HR-HPV infection. Among HIV-negative MSM (Table 2), reporting ≥ 50 lifetime sexual partners (aOR 3.809, p = 0.042) was the only factor significantly associated with anal HR-HPV infection.

### Factors associated with any HPV infection and infection by multiple HPV genotypes

A similar multivariable analytic approach was then used to assess associations of any anal HPV infection and infection by multiple anal HPV genotypes among Toronto MSM.

In a combined cohort analysis that included both HIV infected and uninfected participants, HIV serostatus was the only variable significantly associated with any anal HPV infection (aOR 2.260; 95% CI 1.261–4.049; p = 0.006; Table 3). Limiting this analysis to HIV infected participants, self-reported RAI in the last 6 months was the only variable associated with HPV infection (aOR 2.683; 95% CI 1.045–6.890; p = 0.040); none of the variables remained significantly associated with any HPV infection among HIV-negative participants.

**Table 3:** Multivariable analysis of risk factors for any anal HPV infection. *aOR *adjusted odds ratio, *CI *confidence interval, Hosmer–Lemeshow goodness-of-fit statistics, p = 0.722.

	aOR	95% CI	p value
**All participants**			
Receptive anal intercourse in the last 6 months	1.871	0.994–3.521	0.052
Current smoking	1.307	0.636–2.689	0.466
≥ 50 lifetime number of sexual partners	1.224	0.459–3.263	0.686
≥ 5 number of sexual partners in the last 6 months	1.416	0.707–2.837	0.327
Use of methamphetamine/ecstasy/MDMA in the last 6 months	1.555	0.631–3.828	0.337
Drug use within 2 h of sex	1.174	0.591–2.334	0.646
HIV status	2.260	1.261–4.049	**0.006**
**HIV-positive participants**			
Receptive anal intercourse in the last 6 months	2.683	1.045–6.890	**0.040**
Current smoking	1.053	0.366–3.033	0.923
≥ 50 lifetime number of sexual partners	0.998	0.198–5.026	0.998
≥ 5 number of sexual partners in the last 6 months	1.718	0.595–4.955	0.317
Use of methamphetamine/ecstasy/MDMA in the last 6 months	0.810	0.254–2.583	0.722
Drug use within 2 h of sex	1.237	0.465–3.292	0.670
**HIV-negative participants**			
Receptive anal intercourse in the last 6 months	1.083	0.428–2.743	0.867
Current smoking	1.617	0.559–4.674	0.375
≥ 50 lifetime number of sexual partners	1.327	0.348–5.056	0.679
≥ 5 number of sexual partners in the last 6 months	1.281	0.469–3.495	0.629
Use of methamphetamine/ecstasy/MDMA in the last 6 months	5.000	0.971–25.751	0.054
Drug use within 2 h of sex	0.959	0.346–2.657	0.936

P value of < 0.05 was determined as statistically significant (bolded).

The prevalence of anal infection by multiple HPV genotypes among HIV-infected participants was 67.3% (191/284), compared to 40.7% (59/145) among their HIV-uninfected peers. In the combined cohort analysis, HIV status (aOR 2.809; 95% CI 1.789–4.412; p = 0.000007), RAI in the last 6 months (aOR 1.899; 95% CI 1.183–3.051; p = 0.008), current smoking (aOR 1.805; 95% CI 1.056–3.086; p = 0.031) and reporting ≥ 50 lifetime sexual partners (aOR 3.295; 95% 1.378–7.881; p = 0.007) all remained significantly associated with anal infection by multiple HPV genotypes (Table 4). Within the HIV-positive population, only RAI in the past 6 months remained associated with anal infection by multiple HPV genotypes (aOR 2.438; 95% CI 1.323–4.494; p = 0.004); among HIV uninfected participants only current smoking remained associated (aOR 2.612; 95% CI 1.018–6.701; p = 0.046). The most common genotypes detected in participants with anal infection by multiple HPV genotypes were HPV16 (41.9%), HPV11 (26.7%) and HPV18 (23.6%) among HIV-infected participants, while HPV16 (40.7%), HPV6 (23.7%) and HPV70 (18.6%) predominated in HIV-uninfected participants.

**Table 4:** Multivariable analysis of risk factors for anal infection with multiple HPV genotypes. *aOR *adjusted odds ratio, *CI *confidence interval, Hosmer–Lemeshow goodness-of-fit statistics, p = 0.878.

	aOR	95% CI	p value
**All participants**			
Receptive anal intercourse in the last 6 months	1.899	1.183–3.051	**0.008**
Current smoking	1.805	1.056–3.086	**0.031**
≥ 50 lifetime number of sexual partners	3.295	1.378–7.881	**0.007**
≥ 5 number of sexual partners in the last 6 months	0.839	0.507–1.390	0.496
Use of methamphetamine/ecstasy/MDMA in the last 6 months	1.539	0.852–2.781	0.153
Drug use within 2 h of sex	1.141	0.689–1.891	0.609
HIV status	2.809	1.789–4.412	**0.000007**
**HIV-positive participants**			
Receptive anal intercourse in the last 6 months	2.438	1.323–4.494	**0.004**
Current smoking	1.461	0.747–2.855	0.268
≥ 50 lifetime number of sexual partners	2.574	0.846–7.832	0.096
≥ 5 number of sexual partners in the last 6 months	0.703	0.368–1.345	0.287
Use of methamphetamine/ecstasy/MDMA in the last 6 months	1.862	0.871–3.982	0.109
Drug use within 2 h of sex	0.862	0.451–1.718	0.709
**HIV-negative participants**			
Receptive anal intercourse in the last 6 months	1.314	0.587–2.941	0.507
Current smoking	2.612	1.018–6.701	**0.046**
≥ 50 lifetime number of sexual partners	4.796	0.958–24.012	0.056
≥ 5 number of sexual partners in the last 6 months	0.972	0.423–2.233	0.946
Use of methamphetamine/ecstasy/MDMA in the last 6 months	1.363	0.512–3.630	0.535
Drug use within 2 h of sex	1.662	0.723–3.823	0.232

P value of < 0.05 was determined as statistically significant (bolded).

## Discussion

This cross-sectional study of MSM living in Toronto, Canada was performed to characterize the anal HPV genotype distribution and risk factors for anal HR-HPV infection and found a high prevalence of both HPV and HR-HPV infection in the anal canal. In particular, the rates of HR-HPV infection in both HIV-positive and HIV-negative MSM were broadly similar to rates seen in other countries such as Spain, Italy and China^25–27^. Factors significantly associated with HR-HPV infection were HIV serostatus, cigarette smoking and a higher number of lifetime sexual partners. After stratifying for HIV status, lifetime sexual partners only remained significant among HIV-negative MSM. Interestingly, although over 60% of participants tested positive for at least one HR-HPV genotype included in the nonavalent vaccine, none of the study participants were infected by all nine genotypes. Even when we narrowed down our definition of “vaccine types” to those genotypes included in the quadrivalent vaccine, only three individuals tested positive for all four vaccine genotypes. While the cross-sectional nature of our study precludes us from determining whether prior HPV infections and subsequent clearance occurred in our cohort, it is important to note that even a prior, natural infection may not necessarily confer immunity. As such, our findings imply that older (median age = 45 years) MSM may still obtain benefit from HPV vaccination.

The risk factors for HR-HPV anal infection identified in our study are consistent with findings from other groups. For instance, cigarette smoking has consistently been associated with anal HPV infection^28,29^, and we also found it to be a risk factor associated with both having any anal HR-HPV infection and having multiple HPV infections. We also found having ≥ 50 lifetime sexual partners to be a risk factor for HR-HPV infection only in HIV-negative MSM, which is in line with data from other groups. For example, a group in China found that sexual behavior such as condomless RAI was associated with anal HPV infection in HIV-negative but not HIV-positive MSM^30^. Likewise, researchers from the Netherlands found that having a higher number of lifetime male sex partners was significantly associated with anal HR-HPV infection in HIV-negative but not HIV-positive MSM^31^. Similarly, studies that largely (> 96%) included HIV-negative MSM have observed that number of recent sexual partners is associated with anal HPV infection^32,33^ or that a high number of lifetime sexual partners and recent RAI are associated with the persistence of anal HR-HPV infection^34^. The reasons for the role of sexual activity in increasing anal HR-HPV infection risk in HIV-negative but not HIV-positive MSM are unclear and merit further investigation.

We decided to focus on the risk factors associated with anal HR-HPV genotypes, and not solely with anal HPV16 infection, given relatively high prevalence of several other HR-HPV genotypes. According to a recent meta-analysis, the prevalence of the following anal HR-HPV infections among HIV-positive MSM was above 5% and even exceeding 15% in some cases: HPV18, 31, 33, 35, 39, 45, 51, 52, 56, 58, 59 and 68. Similar trends with a generally lower prevalence were seen in HIV-negative MSM with rates above 5% for the following anal HR-HPV genotypes: HPV18, 39, 51 and 52^35^. Indeed, while HPV16 was the most frequently found anal HR-HPV genotype in both HIV-positive and HIV-negative MSM in our cohort, prevalence of other HR-HPV genotypes targeted by the nonavalent vaccine were found at high rates up to 16.3% and 8.1% in HIV-positive and HIV-negative MSM, respectively. Moreover, while others have reported a significantly higher prevalence of HPV31, 52 and 58 in HIV-positive compared to HIV-negative MSM^36^, they were found at comparable rates in our cohort; our results highlight the need to broaden our scope of understanding to include all HR-HPV genotypes in MSM regardless of HIV status.

The cross-sectional design of our study is a limitation since it precludes us from making definitive conclusions about the time course of HPV infection detected in our cohort. Notably, several groups have reported a high incidence of HR-HPV infection in HIV-positive MSM with a history of recent sexual activity^37,38^. However, given that recent sexual activity including having a new sexual partner or RAI in the past 6 months did not associate with anal HPV infection, it is likely that the majority of HPV infection from this study reflected a persistent, rather than an incident, infection. This is an important distinction as persistence of HR-HPV infection is a better surrogate marker of anal pre-cancer and/or cancer than the incidence/prevalence, which may reflect a transient infection. Data regarding participants’ HPV vaccination history were not available. However, at the time this study was performed, the HPV vaccine was not yet covered in Ontario for boys or gay men under the age of 26 years. Therefore, although it is unlikely that many of our participants would have received the HPV vaccine, it is not possible for us to completely discount the potential effect of HPV vaccination on the prevalence of HPV genotypes in MSM from Toronto.

In summary, the prevalence of anal HPV and HR-HPV infection was high in a cohort of older MSM in Toronto, Canada, but no participant tested positive for all genotypes targeted by the nonavalent vaccine. Risk factors associated with HR-HPV infection for the entire cohort were HIV serostatus, smoking and the number of lifetime sexual partners. Our findings will be useful to inform HPV vaccine rollout and HPV prevention strategies in Canadian MSM.

## Methods

### Ethics statement

The study was approved by the Research Ethics Board at the University of Toronto (Toronto, Canada). Research was performed in accordance with the relevant guidelines and regulations. Informed, written consent was obtained from all participants.

### Study population

Self-identified MSM were recruited into a cross-sectional study of the epidemiology of HIV and other co-infections at the Maple Leaf Medical Clinic (MLMC) in downtown Toronto, Canada between September 2010 and June 2012. All self-identified MSM at or over the age of 16 receiving clinical care from MLMC were invited to participate in the study. A high-level overview of the prevalence of sexually transmitted infections in this cohort has been previously described^24^.

### Study protocol and specimen collection

Participants attended a single study visit. They completed a computerized, self-administered ACASI questionnaire (Audio Computer Assisted Self-Interview; Questionnaire Development System (QDS) 2.5, Nova Research Company, Bethesda, USA), which included information about socio-demographics, sexual and medical history. The sections on the ACASI questionnaire included: (1) interview information, (2) socio-economic characteristics, (3) sexual history and behavior, (4) STI, hepatitis and HIV testing, diagnosis and treatment, (5) other health behaviors, and (6) HIV and STI knowledge, beliefs and attitudes. Blood, first-void urine and a self-collected anal swab were collected to perform diagnostics tests against viral and bacterial infections as described in the next section. All specimens were stored at 4 °C until pick-up.

### Laboratory tests

Participants were screened for HIV, herpes simplex virus (HSV)-1/2, cytomegalovirus (CMV), hepatitis C (HCV), hepatitis B (HBV) and syphilis with serology tests using 20 mL of blood collected in the serum separating tube and the acid citrate dextrose (ACD) tube. First-void urine was used to test for *N. gonorrhoeae* and *C. trachomatis* as previously described^24^. For HBV testing, “active HBV infection” was defined as having a detectable HBV surface Ag and “ever infected with HBV” was defined as having undetectable HBV surface Ag but a positive HBV surface or core Ab with no vaccination history. With regards to syphilis testing, non-reactive CMIA test was considered as negative, “active syphilis” was defined by positive reactions from all three tests (CMIA, RPR and TPPA), and “previously treated syphilis” was defined by having reaction to CMIA and TPPA but not RPR.

### HPV genotyping

Participants were instructed to self-collect an anal swab by moistening the CultureSwab polyester-tipped swab (BD) tip with a saline solution, inserting the swab about 2–3 cm into the anus and rotating the swab 10 times in one direction and 10 times the other direction. Self-collected anal swab was collected in PreservCyt Transport Medium (Cytyc Corporation) and used to perform microsphere-based HPV genotyping (Luminex Corporation, Austin, USA) to test for 46 mucosal HPV genotypes at the Toronto Public Health Laboratory^39^. Of note, 13 of the 46 genotypes were defined as being high-risk: HPV-16, 18, 31, 33, 35, 39, 45, 51, 52, 56, 58, 59 and 68. All other genotypes were defined as being low-risk. DNA from anal samples were extracted using the Roche MagNA pure 96 automatic extractor. In brief, samples were spun down and subsequently eluted using MagNA Pure 96 DNA and Viral NA Large Volume Kit (Roche) and the Viral NA Universal Program. HPV DNA extracted from anal swabs was amplified by nested PCR using AmpliTaq Gold DNA polymerase (Perkin-Elmer) with the PGMY primers followed by the GP5+/GP6+ primers. HPV type-specific probes of 30 nucleotides in length located at the L1 gene were used to detect the PCR products by xMAP technology. Hybridization was analyzed using a Luminex Liquid Chip 200 flow cytometer (Qiagen) and the Luminex IS software (Luminex). A fluorescence signal of 100 fluorescent units or higher was determined as the threshold for positivity. Beta-globin negative specimens post-amplification were considered invalid and therefore excluded from our analyses.

### Statistical analysis

Data from the laboratory and the ACASI questionnaire were analyzed using SPSS, version 27 for Mac (SPSS). Wilcoxon–Mann–Whitney test was used for continuous variables being reported with medians and interquartile ranges (IQR), while Chi-square test was performed for categorical variables being reported as frequencies and proportions with 95% confidence intervals (CIs). Factors associated with anal HR-HPV infection were determined using logistic regression. A conceptual framework of anal HPV infection among MSM was constructed based on the literature, to help better understand the different risks and correlates, along with any potential interactions, and to guide the construction of our multivariable model. Multicollinearity was assessed by checking the ‘tolerance’ and ‘variance inflation factor’, and it was determined that none of the variables in the models constructed had ‘tolerance’ or ‘variance inflation factor’ values that indicated multicollinearity. A combination of a priori and a change-in-estimate inclusion of variables were used for our model. The following variables that have consistently been associated with HR-HPV infection were selected a priori regardless of the results from the univariate analyses: history of receptive anal intercourse in the last 6 months, HIV serostatus, lifetime number of male sexual partners and smoking status. The following variables were selected using a change-in-estimate approach, defined as changing the parameter estimate of the primary risk factor variable—receptive anal intercourse—by 10% or higher: number of sexual partners in the last 6 months, use of methamphetamine, ecstasy or MDMA in the last 6 months and drug use within two hours of sexual activity.
